# Benefits of A Three-Day Bamboo Forest Therapy Session on the Psychophysiology and Immune System Responses of Male College Students

**DOI:** 10.3390/ijerph16244991

**Published:** 2019-12-08

**Authors:** Bingyang Lyu, Chengcheng Zeng, Shouhong Xie, Di Li, Wei Lin, Nian Li, Mingyan Jiang, Shiliang Liu, Qibing Chen

**Affiliations:** College of Landscape Architecture, Sichuan Agricultural University, Chengdu 611130, China; beyonglv@163.com (B.L.); zcclandscape@163.com (C.Z.); xieshouhong798@163.com (S.X.); frank0707@126.com (D.L.); landscape1990@163.com (W.L.); nli@sicau.edu.cn (N.L.); jmy@sicau.edu.cn (M.J.); liushiliang9@163.com (S.L.)

**Keywords:** bamboo forest therapy, psychological responses, physiological responses, immune system

## Abstract

Forest therapy is a fast-growing treatment approach, as it has the potential to alleviate stressful life events and to improve psychological well-being and physical health. Bamboo forests are widespread in southwestern China. Nevertheless, a knowledge gap on the specific health benefits of bamboo forest (BF) therapy still exists. To explore the psycho-physiologic responses of participants to the effects of BF therapy, 60 male adults aged between 19 and 24, with similar healthy conditions, were selected to participate in this study. A one-group pretest–posttest design was used for the BF sites and the city site (CS) to compare the difference in the psycho-physiologic responses of participants before and after the test. Participants at the BF sites participated in a three-day bamboo forest therapy session, and those at the CS participated in a three-day urban program. Blood pressure, heart rate, and peripheral oxygen saturation were measured as the physical signs, and the profile of mood state (POMS) questionnaire was completed by the participants for the psychological evaluation. Blood was sampled, and natural killer (NK) activity, the number of NK cells, and the levels of corticosterone, granulysin, perforin, and granzyme A/B in peripheral blood lymphocytes (PBLs) were measured. All the measurements mentioned above were performed at 08:00 on the first and fourth days within the test. Results indicated that the three-day BF therapy was capable of enhancing positive mood states and also reducing negative mood states in the male participants. The blood pressure and heart rates of the male participants decreased, while the peripheral oxygen saturation increased after the three-day BF therapy session. Furthermore, BF therapy significantly increased NK activity and the number of NK cells and perforin-, granulysin-, and granzyme A/B-expressing cells and significantly decreased the corticosterone level in PBLs in the male participants. The three-day BF therapy session improved the psychological and physiological well-being and enhanced the immune functions of the male college students.

## 1. Introduction

As urbanization progresses around the globe, many advantages of developed infrastructures and artificialized urban environments might be closely related to negative health outcomes in modern people [1]. However, given workplace limitations and the appeal of urban life, an increasing number of people prefer to live in large cities rather than rural areas, especially college graduate students. This trend is projected to continue and intensify [2]. As human society becomes increasingly urbanized, various physiological and psychological diseases are caused by stress, thus affecting well-being and health [3]. Research has shown that high blood pressure (BP) costs the U.S. approximately $48.6 billion per year and affects 1 in 3 Americans [4]. In China, the phenomenon of poor health in urban populations is remarkable because the proportion of people with poor health is as high as 76% [5]. College students have several health risk factors, including irregular sleep patterns, personal relationship changes, over-drinking, and academic pressures, and they experience a large amount of stress, anxiety, and depression [6]. Previous studies have shown that approximately 50% of college students experience significant levels of stress, anxiety, or depression, or a combination of these conditions [7]. Evidence has shown that people suffer pressure from the urban environment psychologically and physiologically.

Air pollution, noise pollution, water pollution, work pressure, and other stresses related to urban environments are increasingly compelling people to seek forms of stress relief and healthy lifestyles [8,9]. Because of the negative environmental impacts in urban areas, research on the benefits of immersion in the natural environment is important. The 16th century Swiss-German physician Paracelsus declared the following: “The art of healing comes from nature, not from the physician” [10]. In most people, the relationship between survival and the natural environment is inseparable. Studies have indicated that natural environments have the potential to improve the relationship between stressful life events and psychological well-being and physical health [11,12,13,14].

An immersive forest experience known as ‘forest therapy’ has recently received widespread attention as a novel form of psychological therapy for reducing stress and providing a feeling of relaxation. Forest therapy is a fast-growing treatment approach [15], and researchers have sought to improve the description and evaluation of the relationship between forests and human health [16]. Recent field studies on forest therapy have provided interesting scientific data supporting the hypothesis that physiological indices, such as BP, pulse rate, heart rate variability [3,17], and salivary cortisol concentration [18], were decreased after forest therapy. Moreover, compared with a city setting, a forest therapy program led to a significant increase in parasympathetic nerve activity [19] and lower sympathetic nerve activity [17]. Additionally, some research has shown that compared with the urban environment, forest therapy was capable of enhancing positive mood states and reducing negative mood states as specific psychological responses [20,21,22,23]. In addition, forest therapy trips resulted in an increase in natural killer (NK) cell activity, which was mediated by increases in the number of NK cells and the levels of intracellular granulysin, perforin, and granzymes A/B in peripheral blood lymphocytes (PBLs) [24,25,26].

Bamboo is an important forest type in many countries, especially in East and Southeast Asia and in African countries. It is a versatile and important component of the ecology, culture, and economy of these countries [27,28]. Bamboo is a well-known and the most preferred plant in the Chinese landscape design due to its unique beautiful foliage and fast-growing characteristics. However, to our knowledge, the benefits of bamboo forest (BF) therapy on both psycho-physiological and immune system responses have not been investigated experimentally. We hypothesized that BF therapy would also provide benefits similar to those of forest therapy on psychophysiology and the immune system.

In the current research, we investigated the effectiveness of a BF therapy program on the psychophysiology and immune system of a large sample of male college students through field experiments with a one-group pretest–posttest design. The aim of the present study was to investigate the benefits of a three-day BF therapy session on the psychophysiology and immune system of male college students. Further, the mechanism of interactions between the nervous, endocrine and immune systems in participants after bamboo forest therapy was discussed in the present study.

## 2. Materials and Methods

### 2.1. Subjects and Experimental Sites

**Figure 1:**
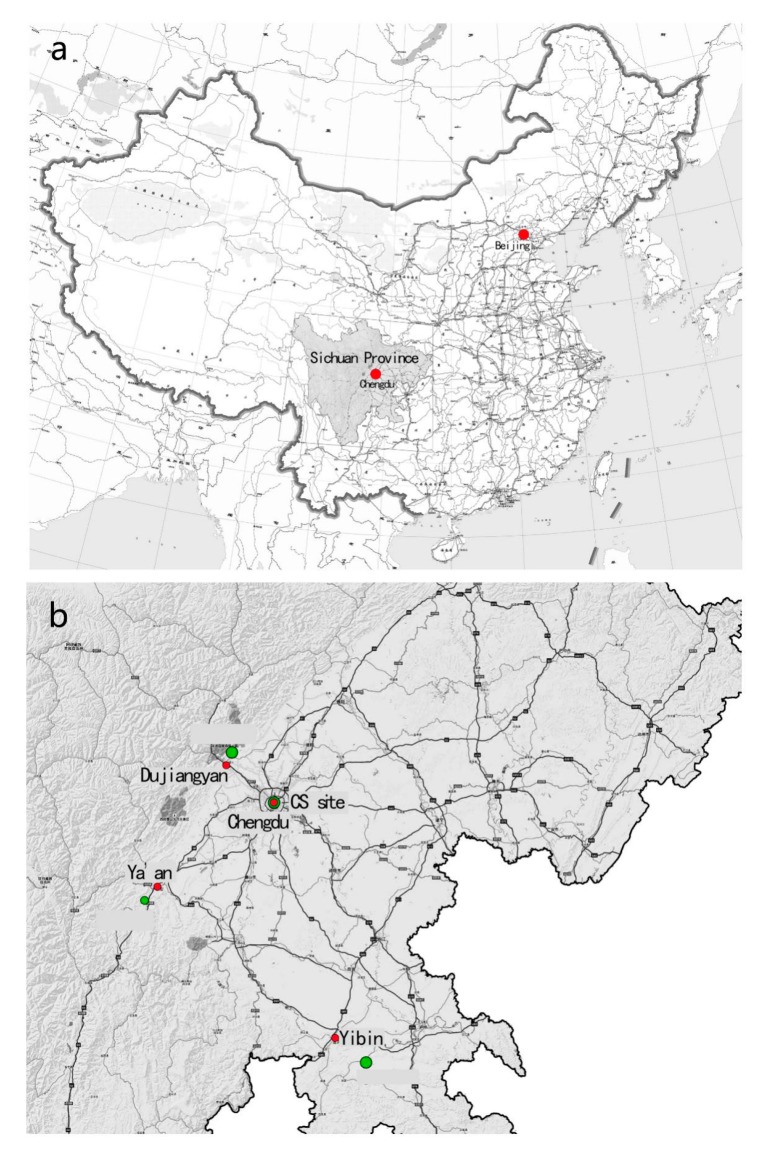
Sketch of experimental sites in the current study. (**a**) Sichuan Province in China and (**b**) the four sites in Sichuan Province. The red points mark the downtown areas and the green points mark the location of each site.

**Figure 2:**
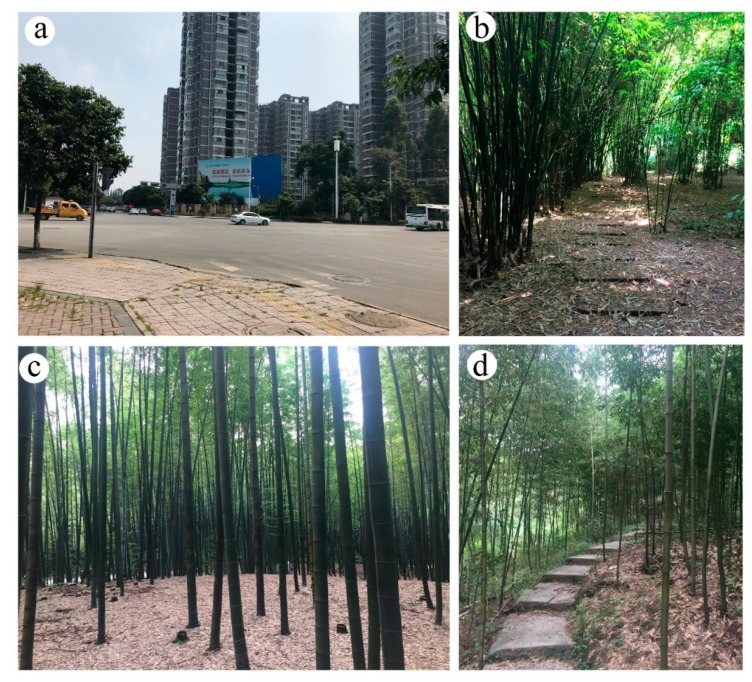
Photographs of the studied sites. (**a**) The city site (CS) site was located at a crossroad in a typical urban environment with cars, buildings, markets, hotels and companies. (**b**–**d**) The bamboo forests sites.

All experimental sites used in this study were located in Sichuan Province in Southwest China. The city site (CS) in Chengdu was located in the center of downtown. In Chengdu, the subjects could view urban buildings, cars, people and other urban elements. For the bamboo forest (BF) sites, a site located near the city of Ya’an was selected. Ya’an is famous for pandas. The dominant forest species of the selected site (and Sichuan Province) is *Neosinocalamus affinis*, a large species of cluster bamboo. The Yibin site was located in Yibin City, which is well known for the Shunan Bamboo Sea, an AAAA-level scenic area as denoted by the Chinese National Tourism Administration. The Shunan Bamboo Sea comprises more than 12,000 ha of large *Phyllostachys heterocycla.* The Dujiangyan site was a bamboo park named ‘zhuhai dongtian’ covered with *Phyllostachys praecox ‘Prevernalis’* in Dujiangyan City. The average height of bamboo forest is 24.5 m and the average density is 600 tufts per hectare. Figure 1 shows the locations of the four research study sites in Sichuan Province in our study, and Figure 2 shows photographs of the four sites.

In this study, 60 male college students from Sichuan Agricultural University participated in three-day field experiments. None of the participants reported any physiological or psychiatric disorders in their personal histories. Subjects who smoked or were alcoholic were excluded from this study. Before the experiment, the goal and experimental procedures of the study were explained to the participants, and their informed consent was obtained. This study was reviewed and approved by the Ethics Committee of Sichuan Agricultural University. To control the background environmental conditions, identical single rooms and similar meals were provided to each subject for the duration of the study period. The subjects were randomly divided into four groups, and each group included 15 males. In addition, basic characteristics of the participants, such as the age, height and weight of each subject, were measured. To avoid physiological differences, the systolic blood pressure (SBP), diastolic blood pressure (DBP), heart rate (HR) and blood oxygen saturation levels of each participant were measured. After collecting the basic data of the participants, analysis of variance was used for the data comparison between the CS and BF experimental sites. The results showed no significant differences between the CS and BF experimental sites in the subjects’ age, height, or weight. The results are shown in Table 1.

Noise, air temperature, absolute illumination, relative humidity, radiant heat, negative air ionization and wind velocity were measured at each experimental site. Compared with the city site, the BF sites showed significant differences in temperature, relative humidity, radiant heat, noise, absolute illumination and wind velocity (*p* < 0.05; Table 2).

### 2.2. Procedures

**Figure 3:**
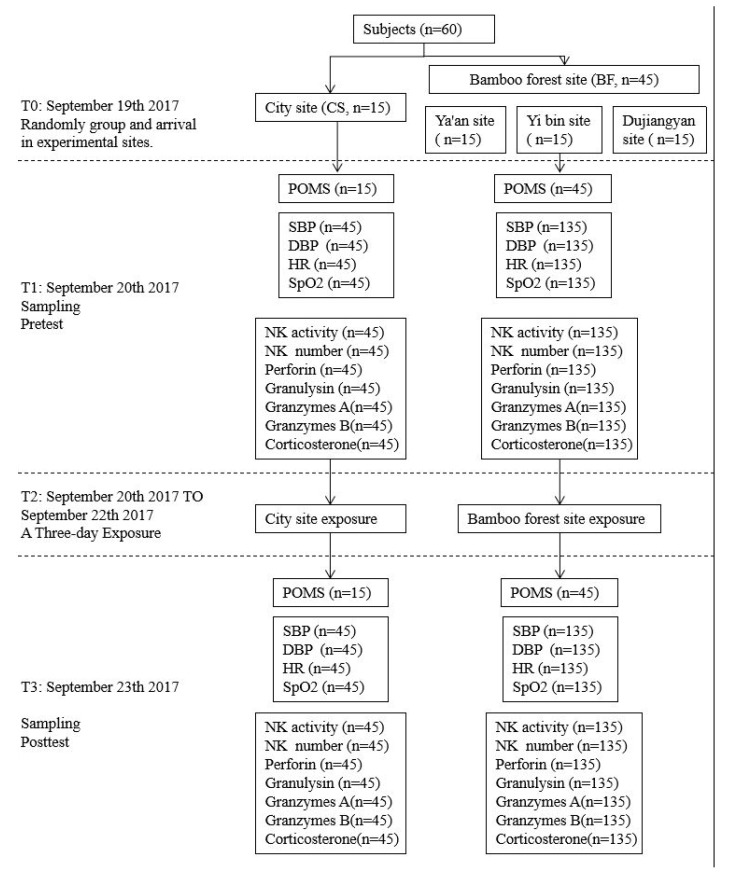
The itinerary for the subjects exposed to the bamboo or urban environment. T0: 19 September 2017; T1: 08:00 on 20 September 2017; T2: 20 September 2017 to 22 September 2017; T3: 08:00 on 23 September 2017.

We chose September for this study, as it is a suitable month for outdoor travel. The weather was sunny during the experimental period. On the afternoon of 19 September, the subjects were divided into four groups. The groups arrived at the arranged hotels near the given experimental sites. The experimental sites were flat areas where the experiment could be easily conducted. The hotels were approximately 300 m from the experimental site. To prevent any effects on emotions, the subjects were allowed to do as they wished in the hotel but were instructed to avoid strenuous exercise and any stimulating activity in their hours of relaxation before sleeping. The experiments of the city site (CS) and bamboo forest sites (BF) were carried out simultaneously. At 08:00 on 20 September 2017 (the first day), psychological and physiological data were taken and blood was sampled. After that, 15 male participants were taken to the city site (CS) and the others were taken to bamboo forest sites (BF). In the next three days, the participants in BF experienced a three-day bamboo forest therapy and the participants in CS were exposed to a city environment. All of the subjects were instructed to remain at their experimental sites from 09:00 to 17:00 except for lunch time. At 8:00 on 23 September 2017 (the fourth day), psychological and physiological data were taken and blood was sampled (see Figure 3).

### 2.3. Measurement

#### 2.3.1. Psychological Indices

A subjective evaluation of mood was performed using the profile of mood state (POMS) questionnaire. The POMS questionnaire is a well-established, analytically derived measure of psychological distress, and its high levels of reliability and validity have been documented. The POMS questionnaire that we used for the analysis was translated to Chinese by Zhu [29]. For this study, the POMS questionnaire included 30 adjectives rated on a 0−4 scale (i.e., 0, not at all; 1, slightly; 2, moderately; 3, substantially; 4, extremely) that could be consolidated into the following six effective dimensions: tension and anxiety (T-A), depression (T-A), anger and hostility (A-H), fatigue (F), confusion (C), and vigor (V). For T-A, D, A-H, F, and C, a lower score represents a better emotional condition. A higher score for V indicates a better emotional condition. The total mood disturbance (TMD) score was calculated using the following Formula (1) [30], and a total of 120 copies of the POMS questionnaires for the four sites provided the data in this study.

(1) TMD score = (T−A) + (D) + (A−H) + (F) + (C) − (V)

#### 2.3.2. Physiological Indices

The physiological indices were measured six times for every participant—three times on the first day and three time on the last day. SBP and DBP were measured with automated BP devices (Omron HEM-7112 Comfort, Omron Health Care (China) Co., Ltd., Dalian, China). BP was measured three times during the intervention, on the left arm with the participants resting in a seated position. Peripheral oxygen saturation (SpO2) was measured with a pulse oximeter (Philips DB18, Philips Medical (Suzhou) Co., Ltd., Suzhou, China) on the index finger of the left hand three times. HR was measured with a single-channel electrocardiograph (Med-ECG-2301, Guangzhou Sanrui Electronic Technology Co., Ltd., Guangzhou, China) three times.

#### 2.3.3. Immune System Indices

Reagents: Roswell Park Memorial Institute (RPMI-1640) medium was purchased from HyClone (Logan, UT, USA). Fetal bovine serum (FBS) was obtained from Clark Bioscience (Richmond, VA, USA). NADΙ was purchased from Sigma (St. Louis, MO, USA), and nitrotetrazolium blue chloride (NBT) was purchased from Biosharp (Hefei, China). Anti-CD3, anti-CD16, and anti-CD56 were purchased from Biolegend (San Diego, CA, USA). The Human CORT ELISA KIT (XL-Eh0551), Human GNLY ELISA KIT (XL-Eh1850), Human Gzms-A ELISA KIT (XL-Eh1375), Human Gzms-B ELISA KIT (XL-Eh1374), and Human PF T ELISA KIT (XL-Eh0770) were purchased from Abcam (Cambridge, MA, USA).

#### 2.3.4. Sample Preparation

Sterile fresh peripheral blood was collected from the study participants and then preserved at −80 °C after adding heparin anticoagulant. Aseptic absorption of 1 mL of anticoagulant peripheral blood was diluted and mixed with the same amount of culture medium. Then, 5 mL of Ficoll solution was first added to a 15 mL centrifuge tube, and diluted blood was gently added to the upper Ficoll layer of two centrifuge tubes. After centrifugation for 20 min at 2000 rpm, the cells at the junction of the uppermost medium were carefully absorbed, and the separator was added to another aseptic centrifuge tube. Then, 5 mL of PBS was added to the centrifuge tube and centrifuged for 10 min at 1500 rpm. The supernatant was removed and then added to the medium for the same cleaning progress. Finally, the cells were divided into two parts—one for lactate dehydrogenase (LDH) detection and one for flow cytometry staining.

#### 2.3.5. LDH and Cell Number Detection

After resuscitation, passage and cryopreservation of K562 cells (K562 cells were the first human immortalised myelogenous leukemia line to be established), K562 target cells in the logarithmic growth period were centrifuged. The collected cells were washed three times with PBS and centrifuged for 5 min at 800 rpm. The cell density was set to 10^5^ for further use. To regulate the concentration of effector monocytes to 10^7^, 100 µL of effector cells and target cells was added to a 96-well plate. Two holes were arranged in each specimen, and the target cell natural release control group (target cell K562 + 1640 medium) and maximum release control group (target cell K562 + NP-40) were prepared at the same time. After incubation for 3 h, 0.1 mL of preheated LDH substrate solution was added, followed by a light-avoiding reaction for 15 min at 37 °C and the addition of 30 µL of 1 mol/L citric acid to the solution. The optical density (OD) value was read at a 570 nm wavelength, and NK activity was calculated as follows: (experimental group OD value−natural group OD value)/(largest group OD value−natural group OD value). The cell numbers were determined by flow cytometry with the default parameters.

#### 2.3.6. ELISA Experiments

Biotin double antibody sandwich enzyme-linked immunosorbent assay (ELISA) was used to determine the levels of corticosterone (CORT), granule lysin (GNLY), granzyme a (Gzms-a), granzyme b (Gzms-B) and perforin (PF). Briefly, the samples to be checked were added to the enzyme-labeled holes, which were precoated with CORT, GNLY, Gzms-A, Gzms-B and PF monoclonal antibody and then incubated. The antibodies against CORT, GNLY, Gzms-A, Gzms-B and PF labeled with biotin were added to bind to streptavidin-HRP to form an immune complex. After incubating and washing, the unbound enzyme was removed, and then substrates A and B were added to generate a blue product, which was converted to final yellow under the action of acid. The depth of color is positively correlated with the concentration of each index in the sample.

Blood was sampled and NK activity was determined; the proportions of NK cells and the levels of granulysin-, corticosterone-, perforin-, and granzyme A/B-expressing cells in PBLs were measured. All measurements were made on the mornings of 20 September and 23 September 2017 at 08:00. All blood samples were placed in an ice/water box at 4 °C, and assays were performed within four hours of the blood draw.

### 2.4. Statistical Analysis

Because the variability between individuals in the physiological and immune system indices was large, we did not compare the difference in the data between the BF sites and CS locations. Rather, differences in all the data before and after each type of trip were compared in the present study. The BF data were the average of the data from the Ya’an, Yibin and Dujiangyan sites. A paired *t*-test was used to compare the data between pretest and posttest after the three-day bamboo forest therapy session. The Statistical Package for Social Sciences software (v20.0, SPSS Inc., Chicago, IL, USA) was used for all statistical analyses. All data are presented as the means ± standard errors (SEs), and differences were considered significant at *p* < 0.05.

## 3. Results

### 3.1. Bamboo Forest Therapy Contributes to the Regulation of Psychological Responses

**Figure 4:**
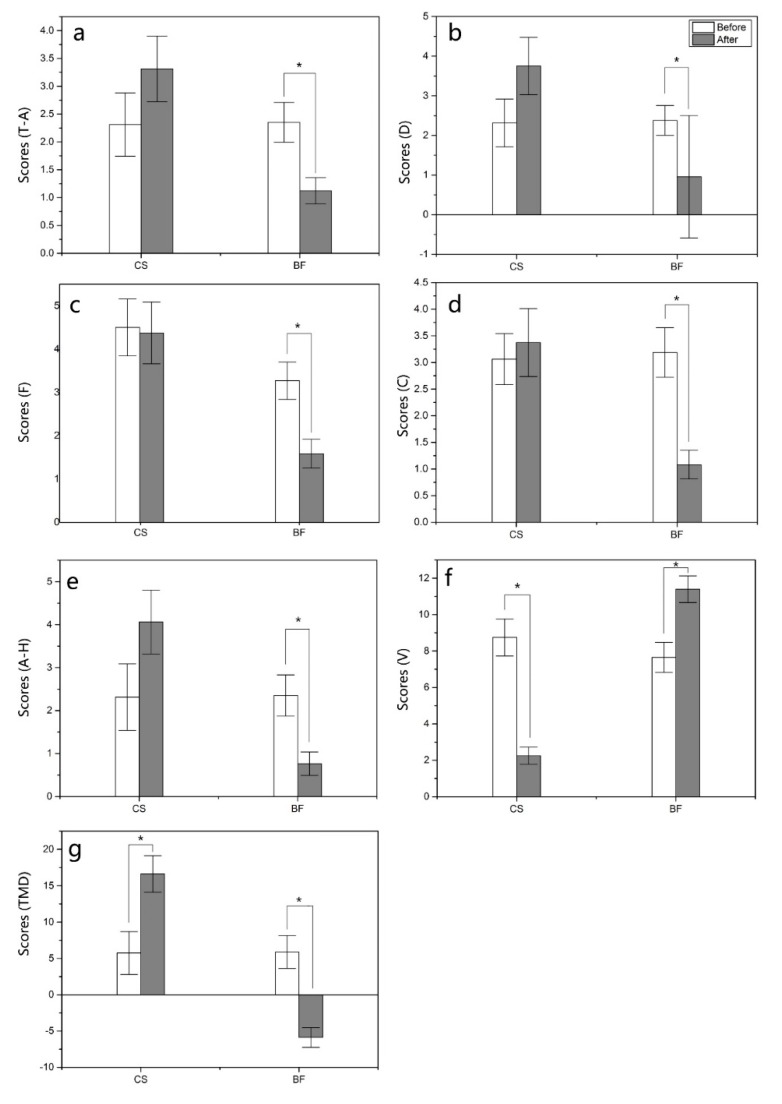
(**a**) tension-anxiety (T-A), (**b**) depression (D), (**c**) fatigue (F), (**d**) confusion (C), (**e**) anger-hostility (A-H), (**f**) vigor (V), (**g**) total mood disturbance TMD. T-scores for tension-anxiety (T-A), depression (D), anger-hostility (A-H), fatigue (F), confusion (C), vigor (V) and total mood disturbance TMD on the profile of mood state (POMS) questionnaire after the bamboo forest and urban programs. A paired *t*-test was used to compare the data between pretest and posttest after the three-day bamboo forest therapy session. The data are presented as the mean ± SEs. * *p* < 0.05, significantly different data between the pretest and posttest for the six mood parameters of the POMS questionnaire by paired t test. CS (n = 15), city site; BF (n = 45), bamboo forest sites.

Scores of negative mood for T-A, D, F, C and A-H at the BF sites significantly decreased after the bamboo forest program (*p* < 0.05; Figure 4a–e). No difference was found in the scores of negative mood for T-A, D, F, C and A-H after the urban program. The scores of V mood significantly increased after the BF program and significantly decreased after the urban program (Figure 4f). In addition, the TMD scores significantly decreased after the bamboo forest program and significantly increased after the urban program (Figure 4g).

### 3.2. Bamboo Forest Therapy Decreases BP in Male College Students

**Figure 5:**
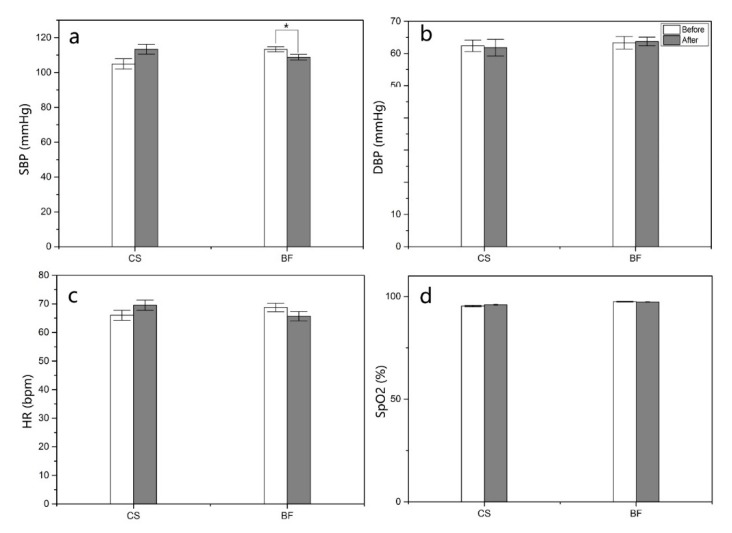
(**a**) systolic blood pressure (SBP), (**b**) diastolic blood pressure (DBP), (**c**) heart rate (HR), (**d**) peripheral oxygen saturation (SpO2). Comparison of systolic blood pressure, diastolic blood pressure, heart rate and peripheral oxygen saturation in participants after the bamboo forest and urban program. A paired *t*-test was used to compare the data between pretest and posttest after the three-day bamboo forest therapy session. The data are presented as the mean ± SEs. * *p* < 0.05, significantly different data between the pretest and posttest for the physiological indices by paired t test. CS (n = 15), city site; BF (n = 45), bamboo forest sites. SBP, systolic blood pressure; DBP, diastolic blood pressure; HR, heart rate; SpO2, peripheral oxygen saturation.

SBP was significantly decreased after the three-day forest bamboo therapy (*p* < 0.05; Figure 5a). At both the CS and BF sites, no significant difference was found between before and after the program in the DBP, HR and SpO2 of male participants (Figure 5b–d). However, the average value of HR was lower at the BF sites and higher at the CS than before the program, although the change was not statistically significant (*p* > 0.05). The results indicated that bamboo forest therapy decreased BP in male college students.

### 3.3. Bamboo Forest Therapy Enhances the Immune Response in Male College Students

**Figure 6:**
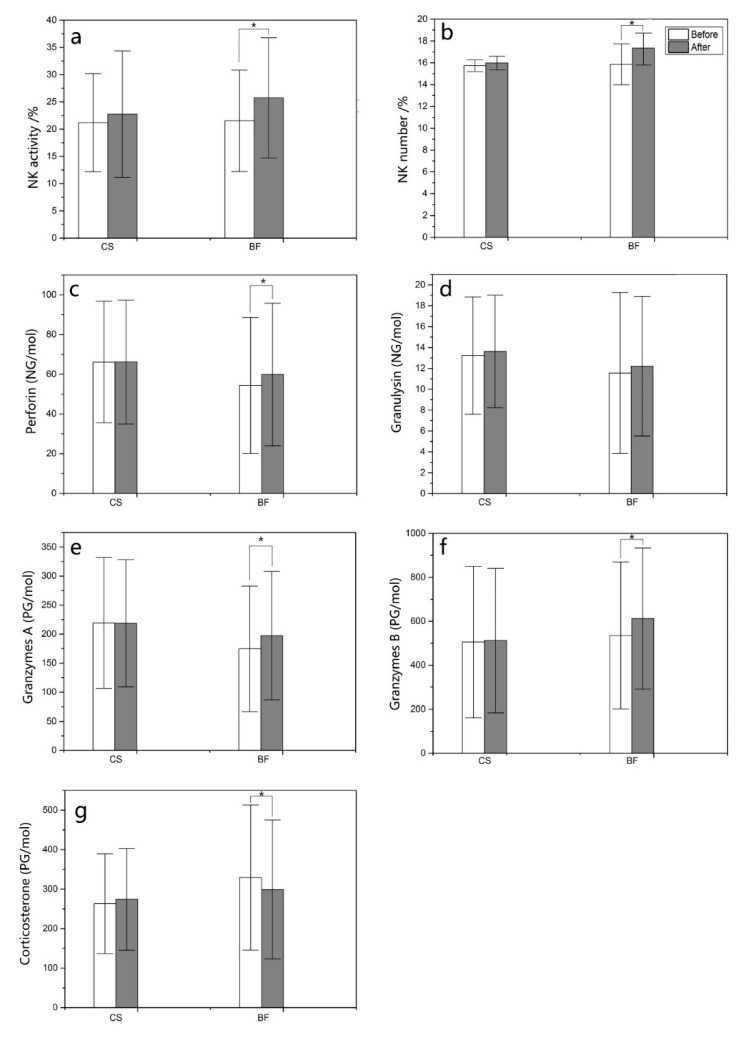
(**a**) NK activity), (**b**) the number of NK cells, (**c**) perforin, (**d**) granulysin, (**e**) granzyme A, (**f**) granzyme B, (**g**) corticosterone. Comparison of NK activity, the number of NK cells, perforin-, granulysin-, granzyme A/B- and corticosterone-expressing cells in peripheral blood lymphocytes in participants after the bamboo forest and urban programs. A paired *t*-test was used to compare the data between pretest and posttest after the three-day bamboo forest therapy session. The data are presented as the mean ± SEs. * *p* < 0.05, significantly different data between the pretest and posttest for the parameters by paired t test. CS (n = 15), city site; BF (n = 45), bamboo forest sites.

Compared with the pretest values, NK activity, the number of NK cells, and the levels of perforin, granzyme A and granzyme B in the PBLs of participants were significantly increased after the BF program (*p* < 0.05; Figure 6a–f). Corticosterone in PBLs was significantly decreased after the BF program (Figure 6g). For the CS program, no significant difference was found in NK activity, the number of NK cells, or the levels of perforin, granulysin, corticosterone, granzyme A and granzyme B in PBLs. In addition, no significant difference was found in granulysin in PBLs with the BF program.

## 4. Discussion

This study investigated the psychological, physiological and immune system effects of a three-day BF therapy on male college students, as well as the difference in these effects before and after the three-day BF therapy.

The present study confirmed that the three-day therapy session in the BF enhanced positive mood states and reduced negative mood states. A previous study reported that forest environments improve psychological well-being [20,21]. Furthermore, the literature has also reported that forest therapy can reduce negative psychological symptoms and increase positive mood states [3,23], which was consistent with our results for the psychological response to BF therapy. 

Physiological data from this field experiment showed that the SBP was significantly lower in participants after a three-day BF therapy session. The DBP and HR exhibited the same trend as SBP, but the change was not statistically significant. The SpO2 of participants was higher after the BF program than before the program. However, no difference in physiological indices was found between pretest and posttest with the urban program. Previous studies have also shown that the mean HR was significantly lower when participants viewed a forest area than when they viewed an urban area [23,31,32,33]. The present result on the physiological benefits of BF therapy is partly consistent with the previous study. BP is one of the vital signs, along with respiratory rate, HR, SpO2, and body temperature. Normal resting BP in an adult is approximately 120 mm of mercury systolic and 80 mm of mercury diastolic. SpO2 is the fraction of oxygen-saturated hemoglobin relative to total hemoglobin in blood. Normal blood oxygen levels in humans are considered 95%–100%. Given the probable differences between individuals, the decrease in DBP and HR and the increase in SpO2 between the pretest and posttest measurements were not statistically significant. However, despite these differences between individuals, it seems that there is a relatively clear tendency showing the beneficial effects of BF therapy sessions compared with urban environment sessions on the physiological response of male college students.

The present study provided evidence for strengthening of the immune system in male college students after a three-day BF therapy session. The results of a paired *t*-test comparing the pretest and posttest conditions showed a significant increase in NK activity, the number of NK cells, and the levels of perforin and granzymes A/B in lymphocytes and a significant decrease in corticosterone in the blood samples. Recent field studies on forest bathing have provided interesting scientific data indicating that forest bathing trips significantly increased NK activity, the number of NK cells, and the levels of perforin, granulysin, and granzymes A/B in PBLs, and the increased NK activity lasted for more than 7 days after the trip [24,25,26]. Other studies have shown that participants in a two-day forest therapy session showed a significantly larger increase in NK cell activity than participants in the control group [34]. In addition, a forest bathing trip can increase NK activity, and this effect is at least partially mediated by increasing the number of NK cells and by the induction of intracellular anticancer proteins. However, no significant difference was found between pretest and posttest in the urban program in NK activity, the number of NK cells, the levels of granulysin, perforin, granzymes A/B in lymphocytes, or corticosterone levels. NK cells are a sub-population of lymphocytes that are able to recognize and lyse a wide variety of target cells [35]. NK cells are recognized as a separate lymphocyte lineage, with both cytotoxicity and cytokine-producing effector functions [36]. Recent studies continue to confirm the importance of NK cells for host resistance, particularly against tumor metastasis [37] and against infection by certain viruses [38]. One mechanism for NK cells to induce tumor- or virus-infected target cell death involves granule exocytosis, with the direct release of cytolytic granules containing perforin, granzymes and granulysin that kill target cells via apoptosis [39]. The increase in the number of NK cells and perforin-, granulysin-, and granzyme A/B-expressing cells in PBLs represents benefits for humans [24]. Therefore, bamboo forest therapy enhances the immune function of male college students by increasing NK activity, the number of NK cells and the levels of intracellular granulysin, perforin, and granzymes A/B in PBLs.

BF therapy can improve the psychological and physiological well-being and enhance the immune functions of male college students. However, the mechanism underlying the benefits to the psycho-physiological and immune systems after BF therapy is unclear.

First, according to biophilia and human evolutionary theories [40,41], the result may be partly explained by the fact that humans have spent many thousands of years adapting to the natural environment and possess an innate tendency to seek connections with the natural environment and other forms of life. According to Kaplan’s [42] “Attention restoration theory,” an environment that possesses a restorative effect requires four properties: being away, fascination, extent, and compatibility [33]. Consequently, Kaplan argued that natural environments are ideal places to restore diminished attentional capacity and provide these elements. In accordance with these theories, a BF is a restorative environment that may improve the psychological and physiological well-being of people.

Second, aromatic volatile substances (phytoncides) extracted from trees may play an important role in the recovery of the immune system. Many studies have shown the meaningful physiological effects of a forest atmosphere on people [17,43,44]. In addition, floral scents can improve mood states and may lead to improvements in emotional health, depression, and memory disorders [45]. These effects are believed to be achieved by inhaling the forest atmosphere, which includes various phytochemicals mainly produced by trees. Li et al. measured NK activity, the percentages of NK and T-cells, and the levels of granulysin-, perforin-, and granzyme A/B-expressing lymphocytes in the blood of participants who were exposed to a room containing many phytoncides produced by vaporizing *Chamaecyparis obtusa* (hinoki cypress) stem oil [46]. Phytoncides, such as α-pinene, β-pinene, β-cadinene, and limonene, were detected in the hotel room air during the experiment. The results showed that phytoncides from trees can increase NK activity, the number of NK cells, and the expression of intracellular perforin and granzyme A/B in male subjects. In addition, essential oils derived from various plants have neuroprotective effects against neurodegenerative conditions in vivo and in vitro [47]. Other studies have also shown that alcohols, acids, alkanes, phenols, and ketones are the main aromatic volatile substances in the BF environment [48,49]. Therefore, phytoncides in BF air may partially contribute to the increased NK activity, the number of NK cells, and expression of intracellular perforin and granzyme A/B in subjects after a three-day BF therapy session.

Third, in addition to the phytoncides, the physical environments at BF sites that differ from the environment at the CS may affect the benefits in participants who experienced a three-day therapy program. A beneficial environment such as a forest, which should include diverse vegetation and ecological components, helps improve the effect of a psychotherapeutic intervention [50,51]. In addition, a five-senses experience from walking or staying in a forest was reported to relieve stress and thus yield health benefits [43]. Additionally, there has been little research on the effects of physical environments on the immune system. Exposure to high concentrations of atmospheric particulate matter, noise and ultraviolet light for a long time is not beneficial for the immune system of humans. However, the urban environment possesses characteristics that are unnatural, unhealthy, uncomfortable, too bright and noisy [19]. In the present study, the absolute illumination at the BF site was significantly lower than that at the CS location. The temperature at the BF site was close to 25 °C, which is a more suitable temperature for humans than the temperatures at the CS location. The noise level at the BF sites was significantly lower than that at the CS location. Based on our field investigation, the environment at the BF sites was significantly different from that at the CS location in terms of absolute illumination, temperature, noise and negative air ionization. Therefore, the physical environments in the BF may induce psycho-physiological relaxation and immune system recovery.

Fourth, the cultural conceptions about bamboo may be an explanation for the positive effects on psychological and physiological responses after a three-day BF therapy session. Mental health and culture are intertwined, and culture is a great determinant of mental well-being and psycho-pathological state. Culture also influences the cause, perception, symptomatology, course, health-seeking behavior and treatment of mental illness [52]. Studies have demonstrated that adaptation to culturally characterized visual environments may lead to distinct patterns of perception [53]. Bamboo culture has a long history and is well known in East and Southeast Asia and in African countries. Bamboo-derived objects are prevalent among people living in bamboo-growing areas, and people have developed a special attachment to bamboo, which has developed into a bamboo culture. In our study, participants who viewed the landscape of BF presented positive patterns of perception that were influenced by bamboo culture.

**Figure 7:**
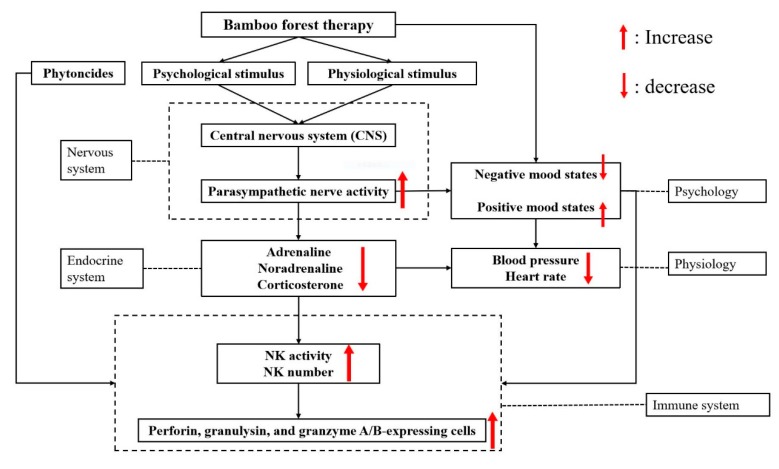
The mechanism of interactions between the nervous, endocrine and immune systems in participants after a three-day bamboo forest therapy.

Evidence-based research has clarified the benefits of forest therapy on psychophysiology and the immune system. However, the mechanism underlying the benefits in participants after a three-day bamboo forest therapy were not clear. Actually, there are complex interactions and exchanges of biologically active molecules between the nervous, endocrine and immune systems. Pathways between the brain and the immune system are bidirectional [54]. Two pathways link the brain and the immune system: the autonomic nervous system and neuroendocrine outflow via the pituitary. The central nervous system (CNS) can communicate with the immune system following activation of the hypothalamic–pituitary–adrenal (HPA) axis and the sympathetic nervous system (SNS). All immunoregulatory processes take place within a neuroendocrine environment that is sensitive to the influence of the individual’s perception of and response to events in the external world. Because lymphocytes bear receptors for various hormones and neuropeptides, the cellular interactions that mediate humoral and cellular immune responses can be modulated by the neuroendocrine environment in which these immune responses occur. In addition, a striking example of CNS involvement in the modulation of immunity is the Pavlovian response, which is a classical conditioning of antibody- and cell-mediated immune responses [55]. Therefore, the interactions between the nervous, endocrine and immune systems could explain the psychophysiology and the immune system responses in participants after a three-day BF therapy session (Figure 7).

Participants were stimulated by the BF environment both psychologically and physiologically. First, the CNS, including the brainstem, had increased parasympathetic nerve activity and suppressed sympathetic nerve activity by neuropeptides after the stimulus of BF therapy [32,56]. The effects on the parasympathetic nervous system could include a reduction in HR, improvements in heart rate variability and baroreflex sensitivity parameters, changes in cytokine expression, or other electrophysiological or central modulations [57]. For forest stimuli, the resulting elevated parasympathetic nervous activity, which is usually observed under conditions of relaxation, indicates that forest bathing may facilitate physiological relaxation [56,58]. In the present study, the decrease in BP, HR, and score of negative mood state may be caused by the activity of the parasympathetic nervous system. The parasympathetic nervous system is also associated with decreases in blood levels of adrenaline, noradrenaline and corticosterone via hormones secreted from the pituitary gland [54]. The levels of corticosterone in PBLs were significantly lower after the three-day BF therapy session but not after the urban program in this study. Forest bathing trips were shown to significantly decrease urine adrenaline and noradrenaline concentrations in males with lower stress [59]. Alternatively, neuroendocrine factors may modulate the immune response indirectly by affecting the production of lymphokines and monokines [60]. Reports indicated that adrenaline and noradrenaline inhibit human NK activity [61,62]. In addition, physical and/or psychological stress decreases NK activity, NK receptor levels, and mRNA transcription levels of granzymes and perforin in mice [63]. Additionally, psychological states are related to the immune system, such that a good psychological condition in participants enhances immunity [64]. Therefore, the improvement in the immune system may be caused by a decrease in adrenaline and noradrenaline concentrations and a positive psychological condition. The increase in parasympathetic nerve activity after a three-day BF therapy session resulted in low levels of adrenaline and noradrenaline and psychological and physiological relaxation. In the urban program, no significant difference in psychophysiological and immune system responses was found between pretest and posttest data in participants.

## 5. Conclusions

A three-day BF therapy session was capable of enhancing positive mood states and reducing negative mood states in male college students. The BP and HR of male college students were decreased, and SpO2 was increased after a three-day BF therapy session. BF therapy significantly increased NK activity, the number of NK cells, and the levels of perforin, granulysin, and granzymes A/B in PBLs in male college students. In summary, a three-day BF therapy session can improve the psychological and physiological well-being and enhance the immune functions of male college students. First, the stimulation of BF therapy increased parasympathetic nerve activity and suppressed sympathetic nerve activity in participants. Then, the concentrations of adrenaline, noradrenaline and corticosterone in the PBLs of participants and stress were reduced after the increase in parasympathetic nerve activity. Finally, a decrease in adrenaline and noradrenaline concentrations and a positive effect on psychology may lead to an increase in NK activity, the number of NK cells, and the levels of perforin, granulysin, and granzymes A/B in PBL.

This study is the first to research the benefits of a three-day bamboo forest therapy session on the psychophysiology and immune system responses of male college students. We gave an explanation of the mechanism underlying the benefits in humans and the interactions between the nervous, endocrine and immune systems in participants after a three-day bamboo forest therapy. However, the study has limitations. A small sample size may be the limitation for the data statistics. Individual differences are also a difficult problem to address. While the benefits of bamboo forest therapy to college students was obvious, aromatic volatile substances (phytoncides) extracted from bamboo forests should be tested. The inclusion of a larger number of participants in this experiment would have produced more scientific and objective results. Further studies with larger samples, including subjects with illnesses such as cardiovascular disease, hypertension and cancer, are warranted.

## Figures and Tables

**Table 1:** Basic information of the sampling subjects at the CS and bamboo forest (BF) sites included in this study. Body mass index (BMI) = weight (kg)/[height (m)]^2^. All data are presented as the mean ± SEs. CS, city site; BF, bamboo forest sites.

Parameter	CS	BF
Sample No. (count)	15	45
Age (years)	21.6 ± 0.34	21.8 ± 0.25
Weight (kg)	65.5 ± 1.23	64.1 ± 0.82
Height (cm)	175.2 ± 0.50	174.6 ± 0.49
BMI (kg m^−2^)	21.3 ± 0.45	20.9 ± 0.24

**Table 2:** Comparison of the environmental factors of the two environmental sites. Data are presented as the mean ± SEs. CS, city site; BF, bamboo forest sites.

Parameter	CS	BF
Temperature (°C)	28.9 ± 0.26	22.9 ± 1.21
Relative humidity (%)	60.5 ± 2.53	81.1 ± 4.23
Radiant heat (°C)	34.5 ± 0.73	23.1 ± 1.52
Noise (dB)	70.1 ± 0.68	45.6 ± 1.21
Absolute illumination (lux)	6585.7 ± 881	1578.3 ± 623.15
Wind velocity (m/s)	0.9 ± 0.19	0.2 ± 0.13
Negative air ionization (ions/cm^3^)	573.3 ±15.08	962.6 ± 38.97
